# California Coastal Upwelling Onset Variability: Cross-Shore and Bottom-Up Propagation in the Planktonic Ecosystem

**DOI:** 10.1371/journal.pone.0062281

**Published:** 2013-05-15

**Authors:** Fanny Chenillat, Pascal Rivière, Xavier Capet, Peter J. S. Franks, Bruno Blanke

**Affiliations:** 1 Laboratoire des Sciences de l'Environnement Marin (LEMAR), CNRS/UBO/IRD/IFREMER, Institut Universitaire Européen de la Mer (IUEM), Plouzané, France; 2 Laboratoire de Physique des Océans (LPO), CNRS/IFREMER/IRD/UBO, Université de Bretagne Occidentale, Brest, France; 3 Laboratoire d'Océanographie et du Climat (LOCEAN), CNRS/UPMC/IRD/MNHN, Institut Pierre Simon Laplace (IPSL), Paris, France; 4 Integrative Oceanography Division, Scripps Institution of Oceanography, University of California San Diego, La Jolla, California, United States of America; National Oceanic and Atmospheric Administration/National Marine Fisheries Service/Southwest Fisheries Science Center, United States of America

## Abstract

The variability of the California Current System (CCS) is primarily driven by variability in regional wind forcing. In particular, the timing of the spring transition, *i.e.*, the onset of upwelling-favorable winds, varies considerably in the CCS with changes in the North Pacific Gyre Oscillation. Using a coupled physical-biogeochemical model, this study examines the sensitivity of the ecosystem functioning in the CCS to a lead or lag in the spring transition. An early spring transition results in an increased vertical nutrient flux at the coast, with the largest ecosystem consequences, both in relative amplitude and persistence, hundreds of kilometers offshore and at the highest trophic level of the modeled food web. A budget analysis reveals that the propagation of the perturbation offshore and up the food web is driven by remineralization and grazing/predation involving both large and small plankton species.

## Introduction

The high biological productivity and fisheries activity of coastal upwelling systems is characterized by long-term (decadal) variability. Correlations of this biological variability in the California Current System (CCS) with North Pacific climate modes such as PDO (Pacific Decadal Oscillation) and ENSO (El Niño Southern Oscillation) [1], [2], [3], [4] have failed to capture the decadal variability of salinity, nutrients and chlorophyll (Chl-a); the mechanisms driving the biological changes remain unclear. The recently introduced NPGO (North Pacific Gyre Oscillation) [5] is the second dominant mode of variability of sea surface height anomalies (SSHa) in the Northern Pacific and is associated with changes in strength of the central and eastern parts of the North Pacific Gyre. Interannual changes in the NPGO have been shown to explain a significant fraction of the long-term variability of salinity, nutrient and Chl-a in the CCS [5], [6], [7].

Variations in the NPGO correlate with changes in the strength and timing of wind-driven upwelling in the CCS. Upwelling in the central and northern CCS exhibits a strong seasonal cycle in response to seasonally varying equatorward winds. At the spring transition – the onset of upwelling – winds become predominantly upwelling-favorable, usually sometime between January and April, with some latitudinal and interannual variability. *Chenillat et al.* [8] showed that a positive phase of the NPGO is characterized by strong alongshore winter winds leading to an early spring transition, with the opposite patterns during a negative NPGO. These changes in the onset of upwelling change the nutrient input and consequent phytoplankton bloom, which in turn influence the rest of the coastal trophic web [9], [10], [11], [12].

Only a few studies have focused on the mechanisms that communicate changes in the timing of the upwelling onset across trophic levels (*e.g.*, *Dorman et al.* [13] for krill; *Ji et al.* [14], and references therein). A bottom-up process can certainly be anticipated, but its expression in time and space and the way it affects trophic links are unknown. Building on new findings about the seasonal expression of the NPGO and its effect on the onset of upwelling [8], we investigate the influence of changes in the timing of the spring transition on the structure and functioning of the California Current planktonic ecosystem. In particular, we examine the cross-shore dependence of the ecosystem response to a time lag in the onset of wind-driven coastal upwelling.

We use a regional hydrodynamic model forced by two synthetic wind climatologies that only differ in winter, corresponding to early or late upwelling onset, such as those typically arising in NPGO+ or NPGO- conditions [8]. This model is coupled to an ecosystem model composed of several phytoplankton and zooplankton size classes. In principle, this model has enough complexity to reproduce the two types of food chains observed in the CCS: a short coastal chain characterized by large organisms, and a longer offshore chain composed of a more diverse set of organisms [15]. This allows us to explore how the perturbation in the onset of upwelling propagates both in space (across shore) [16] and through the food chain.

## Materials and Methods

### Physical model

The hydrodynamic model is the Regional Ocean Modeling System (ROMS), a three dimensional, free-surface, hydrostatic, eddy-resolving primitive equation ocean model based on the Boussinesq approximation and hydrostatic vertical momentum balance [17]. ROMS has been used successfully to model the North Pacific and particularly the CCS dynamics [5], [18], [19], [20], [21]. The configuration of the model is the same as in *Capet et al.* [22], except that the horizontal resolution is 15 km. In the vertical 32 σ-coordinate levels are irregularly spaced with a higher resolution near the surface to adequately resolve the upper ocean physics and ecosystem dynamics. The grid is rotated to follow the general orientation of the California coastline and covers the entire CCS, from the coast to 1000 km offshore and from Baja California (24°N) to Vancouver Island (50°N). The bathymetry is derived from etopo2 (http://www.ngdc.noaa.gov/mgg/global/etopo2.html) following the procedure described by *Penven et al.* [23].

Monthly mean climatologies are used to force the model at the surface and at its lateral boundaries. This is sufficient for our study and consistent with the horizontal resolution of our configuration: the ocean response to synoptic winds can be large within a few tens of kilometers from the shore (succession of upwelling and relaxation phases, sea breeze effects [24]) but this wind variability is not key to the system functioning on regional scales of 100 km or more [18]. It is worth noting here that high temporal resolution atmospheric forcing fields tend to have detrimental coastal biases [20], [22] with possible implications for the system dynamics [25]. Moreover, using climatological forcing simplifies the construction of the two wind seasonal climatologies typical of early and late upwelling onset. To do so, we manipulate a QuikSCAT wind climatology for the period 2000–2008 by rearranging monthly mean fields as described in ***Table 1***. For the two particular rearrangements we have retained, the spring transition indices (the time of year when winds become upwelling favorable, [26]) differ by approximately 3 weeks, and the annual mean upwelling indices differ by 11% [27]. The rearrangements were chosen such that these differences are typical of those found when comparing NPGO+ and NPGO- winter wind conditions off central California [8]. NPGO fluctuations at regional scale are mainly characterized by a modification of winter winds, with no significant heat flux differences, which could be checked using NCEP reanalysis for the period 1958–2008. Thus, we expect our numerical experiments to be relevant to study NPGO impacts on the CCS ecosystem, despite some degree of idealization.

**Table 1:** **Rearrangement of monthly climatological QuikSCAT wind data to build synthetic monthly climatologies representative of early and late upwelling onset.**

New climatology months	Early upwelling onset	Late upwelling onset
December	December QuikSCAT	November QuikSCAT
January	January QuikSCAT	December QuikSCAT
February	March QuikSCAT	December QuikSCAT
March	April QuikSCAT	January QuikSCAT

An equivalent rearrangement was performed for SST. The first column indicates the month of the new climatology, and the second and third columns indicate the corresponding month of the QuikSCAT climatology used to build the early and late upwelling onset climatology, respectively. All the other months in the new climatologies are identical to those of the QuikSCAT climatology.

### Ecological model

The physical model is coupled to the NEMURO (North Pacific Ecosystem Model for Understanding Regional Oceanography) lower trophic level ecosystem model [28], adapted to the North Pacific Ocean. This ecosystem model has been widely used in the CCS [29], [30], [31], [32]. NEMURO consists of 11 state variables: nitrate (NO_3_), ammonium (NH_4_), silicic acid (Si(OH)_4_), a small and a large phytoplankton (PS, representing non-siliceous phytoplankton; PL, representing diatoms), a small, a large, and a predatory zooplankton (ZS, representing microzooplankton; ZL, representing copepods; ZP, representing euphausiids), particulate and dissolved organic nitrogen (PON and DON), and particulate silica (opal). This model is based on two biogeochemical cycles, for nitrogen and silicon. Nitrogen is found in all living compartments (PS, PL, ZS, ZL, ZP), whereas silica is used by diatoms (PL) and transits to its consumers, ZL and ZP. The ratio Si:N is fixed in this model.

Coupled with a realistic hydrodynamical model, this ecosystem model is complex enough to reproduce the cross-shore gradient observed in biological fields, and in particular the existence of two distinct ecosystems: nearshore, a short food chain characterized by large organisms; offshore, a long food chain composed of organisms of every size [15]. As in many studies [33], [34], biogeochemical parameters were adjusted for a constant temperature, chosen as 10°C [30]. The complexity added by temperature dependent processes, was deemed unnecessary in the context of this study, in particular because NPGO has no major impact on temperature off California [5]. We used the phytoplankton parameters tuned for the CCS by *Li et al.* [31]. The zooplankton parameters were designed for the CCS by comparison with other planktonic model studies [29], [33], [34], [35]. We also used a Holling type III formulation for grazing, which is numerically more stable than the default Ivlev formulation used commonly in NEMURO ([36], [37], and references therein). Note that *Li et al.* [32] compared Ivlev and Holling type III grazing formulations in the CCS and showed that they gave statistically indistinguishable results. A compilation of the main biological parameters is given in ***Table 2***. For more details about NEMURO, the reader may refer to *Kishi et al.* [28].

**Table 2:** **Parameters and terms used in NEMURO model.**

Symbol	Definition	Value		Unit	Source
	Parameters for phytoplankton	PS	PL		
V_maxj_	Maximum photosynthetic rate	0.4	1.0	d^−1^	[31]
K_NO3_	Half saturation constant for nitrate	1.0	3.0	mmolN m^−3^	[28]
K_NH4_	Half saturation constant for ammonium	0.1	0.3	mmolN m^−3^	[28]
K_Si(OH)4_	Half saturation constant for silicate	-	4.0	mmolN m^−3^	[31]
ψ	Ammonium inhibition coefficient	1.5	4.6	(mmolN m^−3^)^−1^	[28]
α	Initial slope of photosynthesis-irradiance curve	0.014	0.028	(Wm^−2^)^−1^ d^−1^	[31]
β	Photoinhibition coefficient	0.001	0.008	(Wm^−2^)^−1^ d^−1^	[31]
Mor_P0_	Mortality rate at 0°C	0.0585	0.0290	(mmolN m^−3^)^−1^ d^−1^	[28]

	Pameters for zooplankton	ZS	ZL	ZP		
Gr_max/PS_	Maximum grazing rate on PS at 0°C	1.0	0.2	-	d^−1^	[29], [33], [34], [35]
G_rmax/PL_	Maximum grazing rate on PL at 0°C	-	1.0	1.0	d^−1^	[29], [33], [34], [35]
G_rmax/ZS_	Maximum grazing rate on ZS at 0°C	-	0.5	0.4	d^−1^	[29], [33], [34], [35]
G_rmax/ZL_	Maximum grazing rate on PL at 0°C	-	-	0.8	d^−1^	[29], [33], [34], [35]
K_PS2Z_	Half saturation constant for PS	1.0	1.0	-	(mmolN m^−3^)^2^	[29], [33], [34], [35]
K_PL2Z_	Half saturation constant for PL	-	1.0	1.0	(mmolN m^−3^)^2^	[29], [33], [34], [35]
K_ZS2Z_	Half saturation constant for ZS	-	1.0	1.0	(mmolN m^−3^)^2^	[29], [33], [34], [35]
K_ZL2Z_	Half saturation constant for ZL	-	-	1.0	(mmolN m^−3^)^2^	[29], [33], [34], [35]
ψ_PL_	ZP on PL grazing inhibition coefficient	-	-	4.605	(mmolN^−1^)^−1^	[28]
ψ_ZS_	ZP on ZS grazing inhibition coefficient	-	-	3.010	(mmolN^−1^)^−1^	[28]
Mor_Z0_	Mortality rate at 0°C	0.0585	0.0585	0.0585	(mmolN d^−3^ m^−3^)^−1^ d^−1^	[28]
Alpha_Z_	Assimilation efficiency	0.70	0.70	0.70	-	[28]
Beta_Z_	Growth efficiency	0.30	0.30	0.30	-	[28]

	Other parameters			
g_T_	Temperature coefficient set to 10°C	2	-	[30]
att_w_	Light Extinction Coefficient of Sea Water	0.0554	m^−1^	[31]
att_P_	Self Shading Coefficient	0.0249	mmolN m^−3^ m^−1^	[31]
Nit_0_	Nitrification rate at 0°C	0.03	d^−1^	[28]
VP2N_0_	Decomposition rate at 0°C, PON to NH_4_	0.10	d^−1^	[28]
VP2D_0_	Decomposition rate at 0°C, PON to DON	0.10	d^−1^	[28]
VD2N_0_	Decomposition rate at 0°C, DON to NH_4_	0.02	d^−1^	[28]
VP2Si_0_	Decomposition rate at 0°C, Opal to Si(OH)_4_	0.01	d^−1^	[28]
RSiN	Si :N ratio	1.0	-	[31]
setVP	Sinking velocity of PON	40.0	m d^−1^	[28]
setVO	Sinking velocity of Opal	40.0	m d^−1^	[28]

Bibliographic sources are indicated in the table. References [29], [33], [34], [35] are based on ecosystem models published data that focus on upwelling system.

Initial conditions and monthly-averaged boundary conditions for the biological fields were taken from the high resolution OFES model (Ocean general circulation model For the Earth Simulator [38], [39], [40]) run with a 0.1° horizontal resolution. The OFES model includes a simple ecosystem model (Nutrient-Phytoplankton-Zooplankton-Detritus, or NPZD type) over the whole Pacific domain, integrated numerically from 2000 to 2007. However, the OFES-NPZD model is composed of only 4 biological components, whereas NEMURO has several nutrients, phytoplankters, zooplankters, and detritus. With the guidance of open ocean observations [41], [42], 2/3 (1/3) of OFES phytoplankton was assigned to NEMURO PS (PL), and each zooplankton class of NEMURO received one third of the total OFES zooplankton. Boundary and initial conditions for silicic acid were provided by nitrate profiles taken from the OFES-NPZD model and adjusted by a mean ratio Si:N of about 2.0, in accordance with the Levitus World Ocean Atlas [43].

### Set of simulations

**Figure 1:**
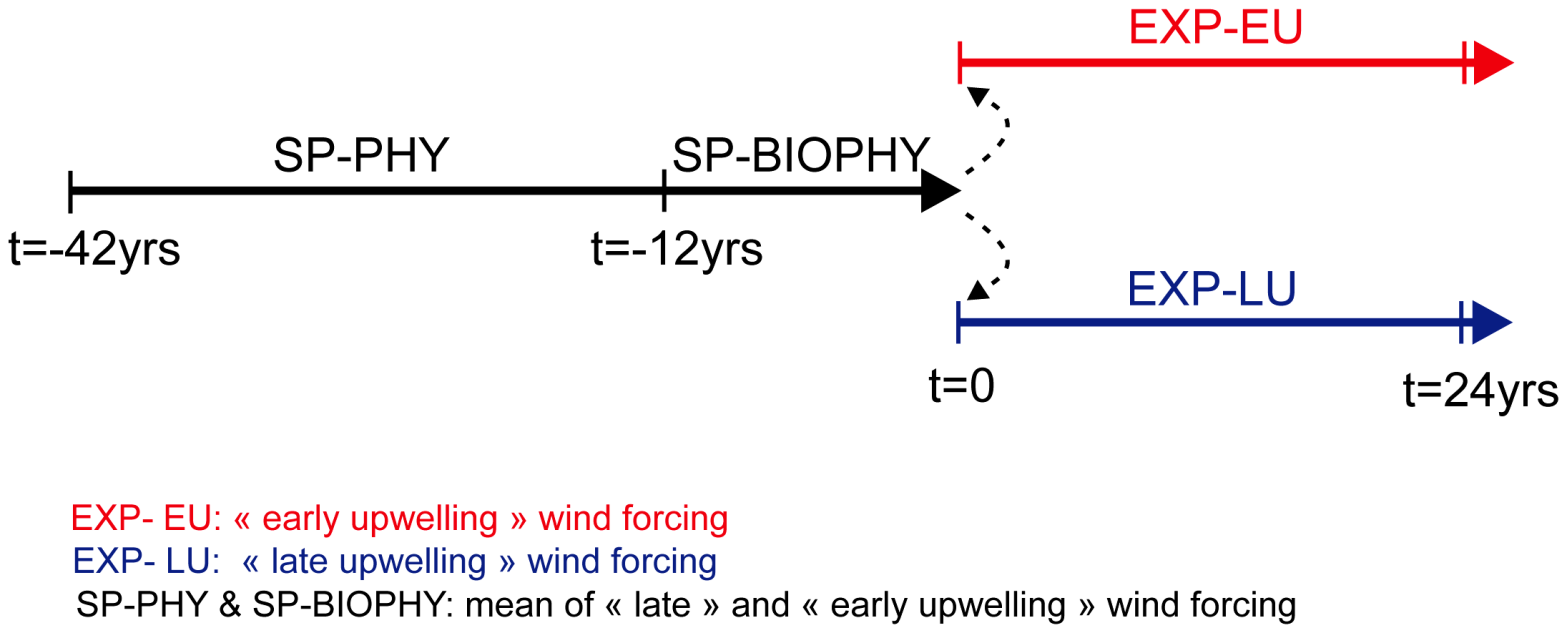
**Experimental layout.** Times are in years. EXP-EU: early upwelling forcing winds. EXP-LU: late upwelling forcing winds. SP-PHY and SP-BIOPHY: mean forcing winds.

A 30-year spin-up simulation was performed with the physical model only (SP-PHY), and we have verified that the end of this run reached a statistical equilibrium. The final state provides an initial condition for the second spin-up that couples biology and physics (SP-BIOPHY). SP-BIOPHY was run for 12 years. These two spin-up simulations were forced by synthetic SST and QuikSCAT wind data given by the average of the early and late upwelling onset climatologies. Next, we ran two 24-year long experiments for the early upwelling onset (NPGO+ like) and late upwelling onset (NPGO− like) scenarios (respectively EXP-EU and EXP-LU). The only differences between these twin experiments were the climatological wind conditions that were representative of the effect of NPGO on winter wind upwelling regime; these two wind fields differ only during wintertime from December to March. The two wind climatologies are based on the study of *Chenillat et al.* [8] and were constructed by combining the original monthly mean wind fields of the QuikSCAT climatology as described in ***Table 1***. The alongshore components of these two wind climatologies differ by 11% off the Central California Current System. The whole spin-up procedure is summarized in ***Figure 1***.

## Results and Discussion

### Model evaluation

We only aim at qualitatively assessing the model skill because our model, forced by climatological atmospheric fluxes, lacks several important features (synoptic forcing variability, resolution of fine scale frontal processes [44], [45]) and because the observations used for comparisons do not span over the same periods.

The evaluation of the model is based on averages calculated over the final 6 years of SP-BIOPHY, except for eddy kinetic energy (EKE) which requires a longer time series for its computation, with high-frequency outputs for velocity. Therefore, EKE is evaluated in a 30-year-long simulation run of the physical model (using the same forcing as SP-PHY).

**Figure 2:**
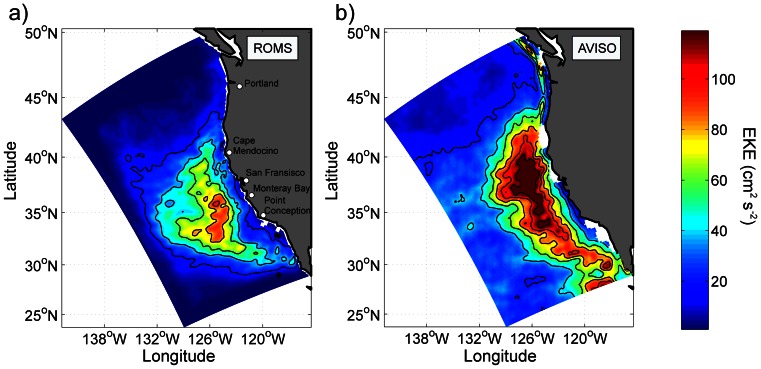
**Annual mean eddy kinetic energy (EKE) from (a) the model and (b) satellite-derived SSH observations (AVISO, 1992–2009).** Units: cm^2^/s^2^. The contour interval is 20 cm^2^/s^2^.

The annual mean climatology EKE is a good proxy for the intensity of mesoscale turbulence. The modeled EKE (***Fig. 2a***) is compared with MADT altimetry data (http://www.aviso.oceanobs.com/duacs/) between 1992 and 2009 (***Fig. 2b***). The model EKE has maximum values around 120 cm^2^/s^2^ in the central CCS, about 300 km from the coast, and minimum values along the coast, similar to the observations. The maximum values of altimetry-derived EKE are higher than the model (200 cm^2^/s^2^) and extend over a broader area, which indicates that the model underestimates EKE. This is a direct consequence of the model resolution that is too coarse to fully resolve mesoscale and submesoscale dynamics in this system [22].

**Figure 3:**
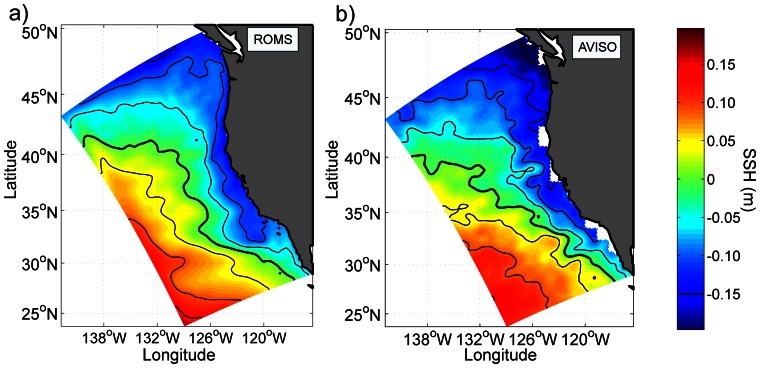
**Annual mean sea surface height from (a) the model and (b) satellite-derived SSH observations (AVISO, 1992–2009).** Units: m. The contour interval is 0.05 m and the 0 m contour is in bold.

To assess the mean surface circulation in the CCS we compare the annual climatological mean model SSH (***Fig. 3a***) with equivalent AVISO data from 1992–2009 (***Fig. 3b***). The cross-shore gradient (from −0.2 m at the coast to +0.2 m offshore) and the placement of the isolines are in fair agreement with the observations, which shows that the intensity of our California Current is realistic. The values nearshore are more negative in the observations than in the model, but altimetric sea level measurement should be used with caution close to shore [46].

**Figure 4:**
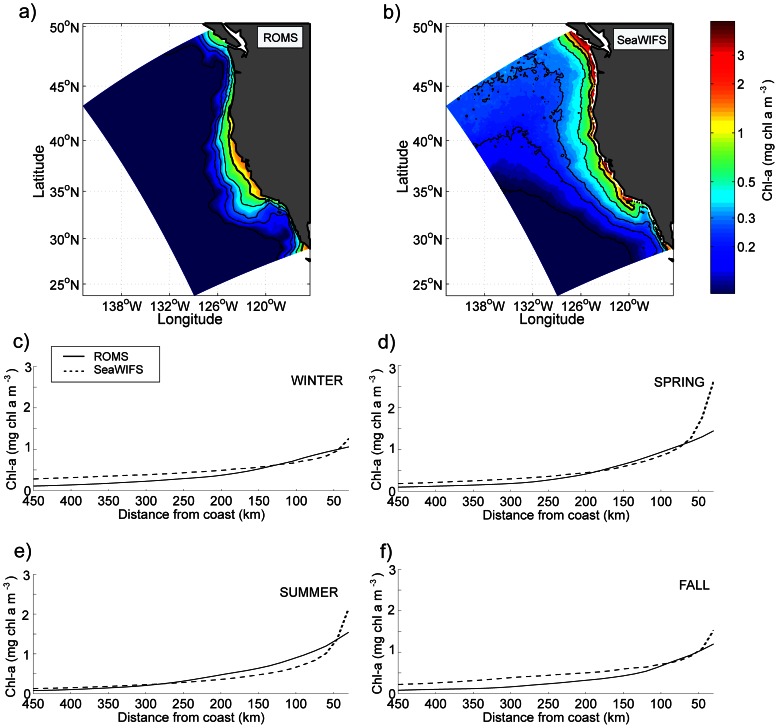
**Annual mean surface Chlorophyll-*a* from (a) the model and (b) observations (SeaWIFS, 1997–2005).** Seasonal cross-shore profiles of surface Chlorophyll-a averaged for the central CCS (from 34.5°N to 40°N) for the model (solid line) and the observations (SeaWIFS, 1997–2005; dashed line) for (c) winter (January–February–March), (d) spring (April–May–June), (e) summer (July–August–September), and (f) fall (October–November–December). Units: mg Chl-a m^−3^.

***Figure 4*** shows the annual climatological mean model Chlorophyll-a (Chl-a) concentration at 10 m depth and climatological Sea-WIFS (Sea-Viewing Wide Field-of-view Sensor) data averaged over 1997–2005. The model phytoplankton biomass (sum of PS and PL, in mmol N m^−3^) is converted to Chl-*a* using a C∶N ratio of 106∶16, and a conversion from C to Chl-*a* based on *Cloern et al.* [47]. The comparison reveals a general agreement between the model and the observations in the central and southern CCS in a 500 km strip along the coast despite a small negative bias of the model very nearshore and far offshore. The main discrepancy is found in the northern CCS with a more systematic negative bias in modeled surface Chl-*a* concentration. Note that our study focuses on central California, where the model best reproduces the observed chlorophyll distribution. Seasonal profiles in the central CCS, averaged from Point Conception (34.5°N) to Cape Mendocino (40°N), confirm the model bias in Chl-a nearshore, mainly in spring (***Figs. 4c–f***). The far offshore negative bias is most prominent in winter and fall, whereas the surface Chl-*a* concentration is in better agreement with observations during spring and summer.

**Figure 5:**
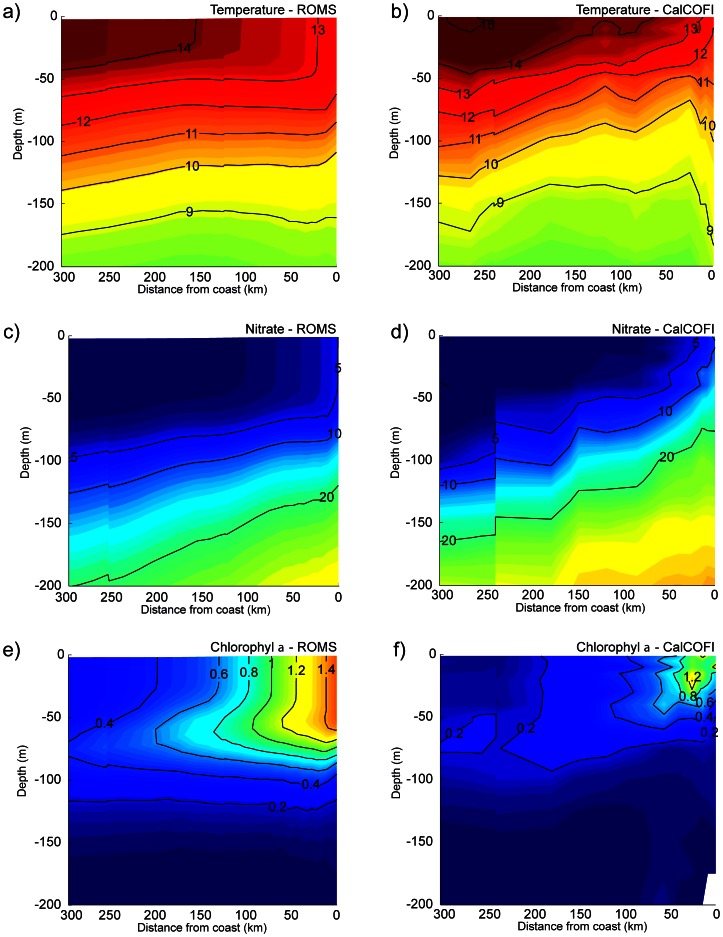
**Comparison along CalCOFI line 70 of mean vertical sections for the model (left) and observations (right):** (a) and (b) temperature (°C); (c) and (d) nitrate concentration (mmol N m^−3^); (e) and (f) Chlorophyll-*a* (mg Chl-a m^−3^).

Finally, we examine the vertical structure of the model (***Fig. 5***) using CalCOFI data averaged over 1949–2000. Along line 70 (south of Monterey), from the coast to 350 km offshore a general agreement is found between model and CalCOFI annual climatological mean temperature, nitrate and Chl-*a* climatologies, including the cross-shore structure of the 13°C isotherm depth (***Figs. 5a–b***), the 20 mmol N m^−3^ isoline (***Figs. 5c–d***), and the presence of a subsurface maximum in Chl-*a* (***Figs. 5e–f***). Some differences are also evident: offshore, the model overestimates Chl-*a* by a factor of 2 at the surface and by a factor of 3 around the depth of the subsurface maximum (70 m). These differences coincide with a model underestimation of subsurface nitrate concentrations. Close to the coast, the model 12°C isotherm is deeper than in the observations, which suggests that the model fails to accurately represent the pathway and/or transformation of upwelling waters.

Overall and despite some weaknesses, the model shows evident skill and realism for both its physical and biogeochemical components. It reproduces a realistic CCS general circulation (for the mean and mesoscale dynamics) and it is able to simulate a seasonal cycle and a spatial cross-shore structure of “bulk phytoplankton” that resemble the observations, at least off central California. In the next section, we analyze the functioning of the modeled biological system and its perturbation by a change in the timing of the onset of upwelling.

### Ecosystem response to differential onset of upwelling

We focus on the central CCS, from Point Conception (34.5°N) to Cape Mendocino (40°N). Diagnoses of tracer concentration and tracer budgets are carried out in two subregional boxes. The nearshore box is bounded by the latitudes 34.5°N and 40°N, the coastline, and a line that follows the mean orientation of the central coast but is located 150 km offshore. The offshore box is defined similarly, but within two lateral limits situated at 300 and 450 km from the coastline. The vertical extension of both boxes is from the surface down to 100 m (or the ocean bottom in areas shallower than 100 m). The ecosystem response will be described in terms of nitrate and biomass concentrations expressed as nitrogen concentrations. We estimate the statistical significance of the differences between the twin resulting climatologies with a non-parametric test [48]. The role of the other components, ammonium and dissolved or particulate nitrogen (involved in the recycling loop), will be discussed in the following sections. For space constraints and clarity, the silicon cycle will be ignored. However, we have verified that, at least in the model we use, it does not play a key role in the ecosystem response to a differential upwelling onset.

**Figure 6:**
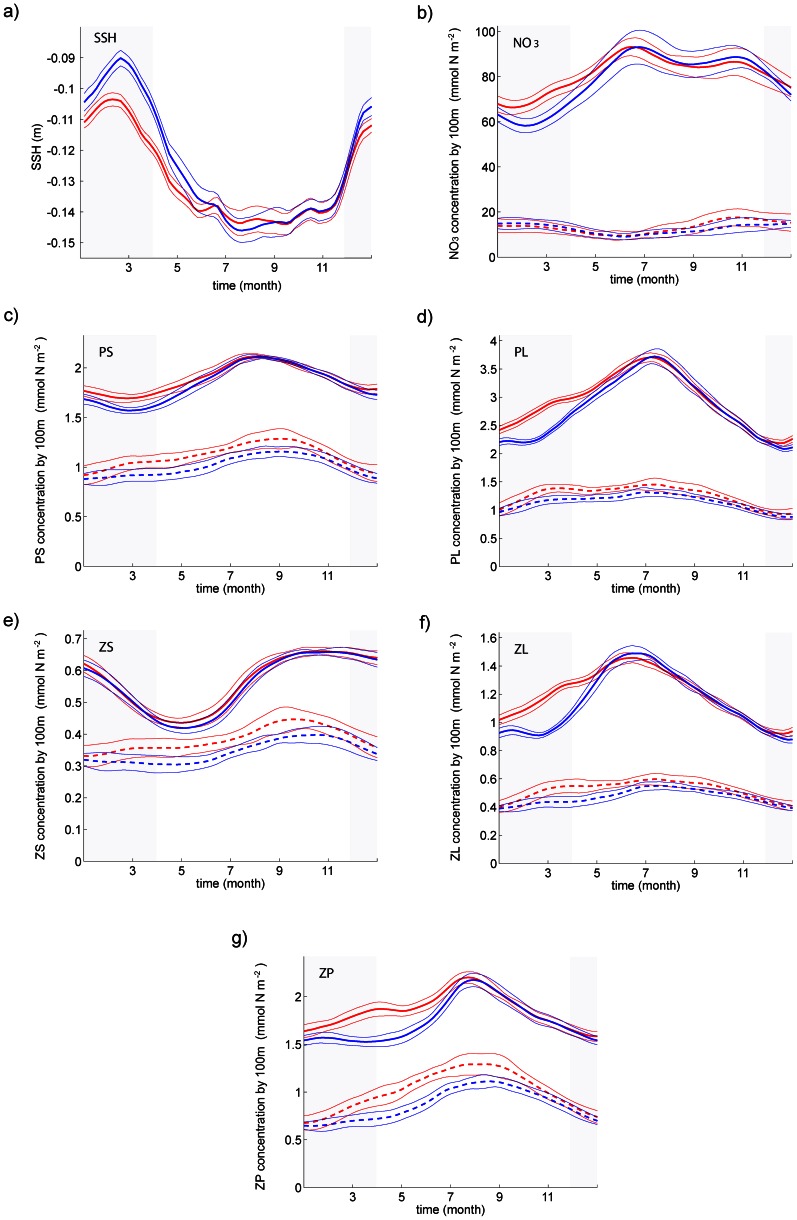
**Time evolution of key modeled variables in the early upwelling (red) and the late upwelling (blue) scenario, in the central CCS.** (a) Nearshore SSH (in m) averaged over a 50 km wide strip along the coast. (b–g) Biological variables (in mmol N m^−2^) integrated from the surface to 100 m depth and averaged over the nearshore region (0–150 km offshore, bold solid line) and over the offshore region (300–450 km offshore, bold dashed line): (b) nitrate; (c) PS; (d) PL; (e) ZS; (f) ZL; (g) ZP. Gray-shadowed areas show the months when winds differ between both scenarios. The thin lines represent the year-to-year variability diagnosed during the 12-year-long scenarios (standard deviation).

For the nearshore domain, the annual mean cycle of all model variables (***Fig. 6***) agrees with the observed central California upwelling dynamics and biogeochemistry, regardless of the details of the winter wind. PL, ZL and ZP (***Figs. 6d, f and g***) dominate the nearshore ecosystem biomass with a marked bloom in late winter/early spring, a peak of biomass in early summer, and a return to lower values afterwards. Their evolution roughly mirrors that of SSH (***Fig. 6a***), but with smoother changes. The peak for ZP (***Fig. 6g***) lags the ZL peak by about a month, and the PL peak by about 2 weeks, in qualitative agreement with a bottom-up forcing of the ecosystem. PS and ZS (***Figs. 6c and e***) are present in much smaller concentrations. The PS bloom appears slightly later (2 weeks) than the PL bloom, and is followed by a smooth peak of ZS in early fall. The evolution of ZS is in quadrature with the evolution of large zooplankton ZL and ZP: its minimum is in spring when the concentrations of the larger plankton size classes increase the fastest, and vice versa in fall.

The mean annual cycles of biogeochemical tracer concentration in the offshore box are 2 to 5 times smaller than in the nearshore box, with a less pronounced (***Figs. 6c*** to ***f***) or even reversed (NO_3_) seasonal cycle (***Fig. 6b***), except for ZP (***Fig. 6g***). The nearshore box minus the offshore box differences are consistent with tracer maxima originating nearshore and moving offshore with some attenuation. Other more subtle differences deal with the biomass of small (***Figs. 6c and e***) and large (***Figs. 6d, f and g***) plankton size classes. Nearshore, the ecosystem biomass is dominated by large plankton species, whereas the offshore trophic chain is longer and consists of a roughly equal proportion of small and large organisms.

We now study the system response to a perturbation in the onset of upwelling from monthly climatologies of the last 12 years of the EXP-LU and EXP-EU simulations.

The modeled sea level seasonal cycle is smooth enough to define the upwelling onset as the time at which the nearshore sea level starts decreasing. This definition gives a statistically significant 10-day difference between the two simulations (***Fig. 6a***). Wind differences also directly translate into a larger annual stock of nearshore nitrate in the “early upwelling” (NPGO+) simulation. A statistically significant difference of 15% is observed during December-April (***Fig. 6b***); the change is only 5% when averaged over the entire year. An impact on plankton nearshore biomasses is observed from January to July. Differences in nearshore biomass of PL (7% in January and 20% in March), ZL (8% and 28%) and ZP (3% and 21%) (***Figs. 6d, f and g***) are consistent with the propagation of the perturbation up the food chain. Interestingly, the small nearshore plankton PS and particularly ZS (***Figs. 6c and e***) are noticeably less affected by the perturbation.

Overall, the winter wind perturbation has a very limited impact on the nearshore ecosystem beyond July. However, a noteworthy consequence of the winter wind perturbation is the presence of a reverse trend in nearshore biological/nutrient concentrations in late summer compared with the beginning of the year. Although the trend is of limited amplitude and tends to be smoothed by spatial averaging, it is apparent for NO_3_ from July to October (***Fig. 6b***). In other words, stronger winter winds lead to reduced nitrogen concentrations about 6 months later in conjunction with a sea level rise (***Fig. 6a***) mainly in July and August. Indeed, the response of a linear eastern-boundary system to periodic winds includes free Rossby waves that would propagate offshore for some months and lead to a rise in sea level [49]. Our non-linear CCS undergoes, on average, a similar SSH evolution and a depression of the upper thermocline (10 to 20 m) that reduces the nutrient input to nearshore areas (not shown).

The effect of the wind perturbation on the offshore ecosystem is distinct and arguably more important. In terms of duration, the increase in biogeochemical concentrations is notable from March to late October, *i.e.*, for most of the year. In particular, the ZP biomass (***Fig. 6g***) is very sensitive to winter winds, with a maximum difference between the two simulations of about 35% in May. Nutrients are a notable exception: they remain strongly depleted throughout the year (***Fig. 6b***).

These results indicate an offshore propagation of the nearshore nutrient input perturbation, in qualitative agreement with the conceptual view of the mixed-layer conveyor model proposed by Botsford et al. [16] (see their Fig. 1). As it propagates offshore at a speed of a few cm/s (not shown), the perturbation also propagates up the trophic chain. We now characterize this cross-shore and bottom-up propagation by studying the functioning of the system by means of tracer budgets.

### Cross-shore difference of biological tracer budget

**Figure 7:**
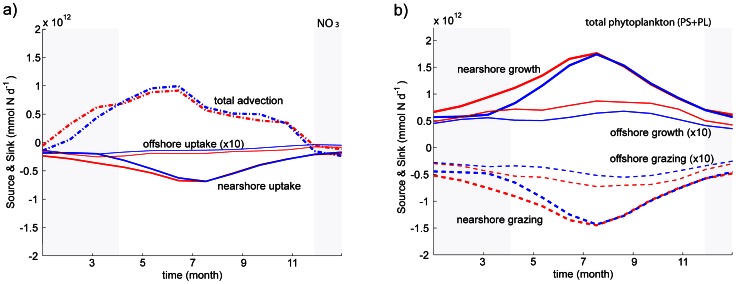
**Time evolution of nitrogen sources and sinks (in mmol N d^−1^) in the early upwelling (red) and late upwelling (blue) scenarios:** (a) nearshore total advection of nitrate, and nearshore and offshore uptake by total phytoplankton; (b) nearshore and offshore total phytoplankton growth and grazing. The offshore fluxes are rescaled by the factor 10 for clarity. Gray-shadowed areas show the months when winds differ between both scenarios.

**Figure 8:**
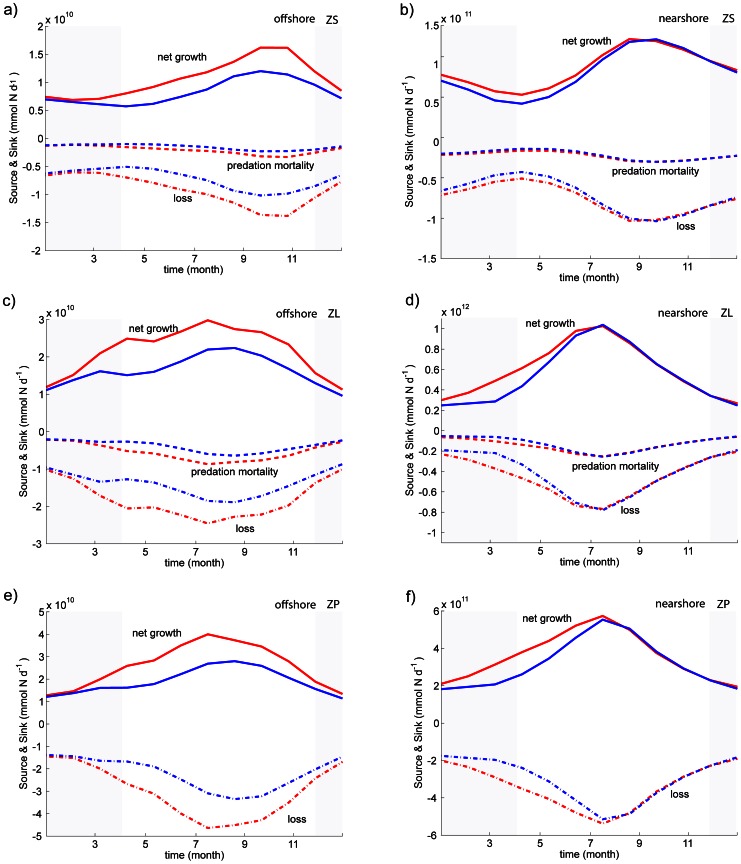
**Time evolution of biological sources and sinks (in mmol N d^−1^) in the early upwelling (red) and late upwelling (blue) scenarios, both nearshore (right) and offshore (left): (a) and (b) small zooplankton (ZS) fluxes; (c) and (d) large zooplankton (ZL) fluxes; (e) and (f) small zooplankton (ZS) fluxes.** Gray-shadowed areas show the months when winds differ between both scenarios.

**Figure 9:**
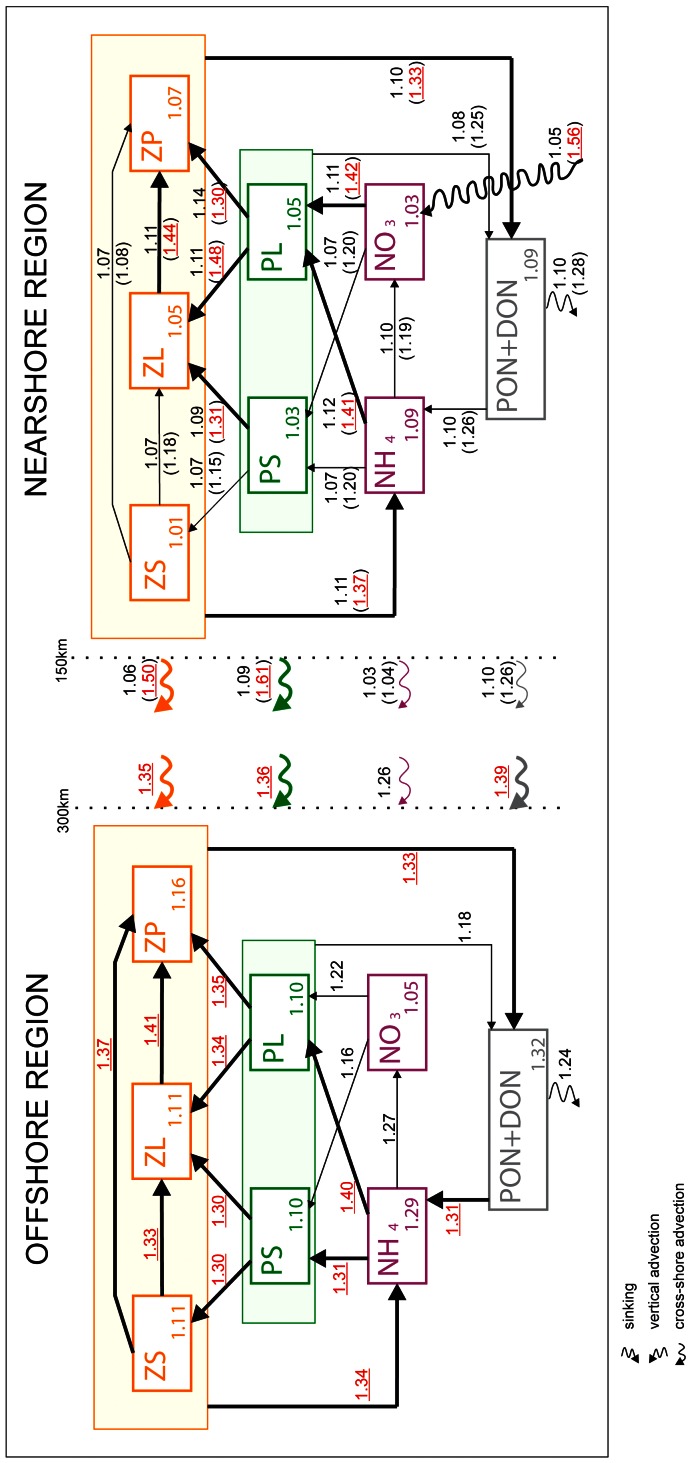
**Biological (biomasses and fluxes) and advective (fluxes) budget for biological tracers in the nearshore and offshore boxes.** Numbers in the main boxes indicate the ratios of the early upwelling scenario annual mean biomasses to the late upwelling scenario annual mean biomasses. Numbers above arrows indicate the ratios of the early upwelling scenario annual means fluxes to the late upwelling scenario annual means fluxes. Note that for the nearshore region, numbers in parentheses indicate the ratios of the winter mean fluxes. Ratios underlined in red above bold arrows emphasize 30% or larger differences between early and late upwelling scenarios.

We analyze the propagation of the effects of the winter wind perturbation in space (from nearshore to offshore) and across trophic levels (bottom-up). We do so by studying the physical and biological terms (flux divergences and source/sink terms) that compose the budget of every biological component of the ecosystem within the box framework defined in section 4. Both seasonal (***Figs. 7*** and ***8***) and 12-year averaged (***Fig. 9***) fluxes are shown. For sake of simplicity, only the most important fluxes are presented.

#### Seasonal biological budgets

The origin of the perturbation can be traced back in the nearshore nitrate budget (***Fig. 7a***): the advection term is larger from January to March under stronger winter upwelling winds. The difference arises primarily from vertical advection (not shown). Over the rest of the year, the nitrate flux divergence remains positive, but some compensation occurs due to the cross-shore (mainly advective) nitrate flux out of the nearshore box in the early upwelling onset case. The advective flux perturbation results mainly in an uptake perturbation: the total phytoplankton uptake increases continuously from January with early upwelling winds, whereas it is constant until the beginning of March with late upwelling winds (***Fig. 7a***). Noticeable nearshore uptake differences are limited to the first half of the year.

We now turn to nearshore bulk phytoplankton production (***Fig. 7b***). We recall that phytoplankton growth is the ombination of new production (nitrate uptake) and recycled production (ammonium uptake). These two processes are not separately shown in ***Figure 7***, but can be easily estimated as follows: new production is reflected by nitrate uptake (***Fig. 7a***), and recycled production is reflected by the difference between phytoplankton growth (***Fig. 7b***) and new production. Approximately half of the nearshore phytoplankton growth is associated with ammonium uptake, *i.e.*, with the recycling loop; the other half is associated with nitrate uptake, *i.e.*, with new production. Moreover, the wind-induced perturbation is of the same order for ammonium and nitrate uptake.

Offshore, total nitrate uptake differences (about 20%) are spread over most of the year (***Fig. 7a***). Unlike the nearshore region, the offshore winter wind-induced perturbation is more important for ammonium uptake than for nitrate uptake. Indeed, the difference between the two upwelling scenarios is about 60% for the total uptake, estimated over the entire year (***Fig. 7b***), which is much more than the 20% difference obtained for nitrate uptake (***Fig. 7a***). Thus, in the offshore region, an early upwelling stimulates recycled production more efficiently than new production.

For the phytoplankton and zooplankton budgets, the consequences of the upwelling onset perturbation are mainly expressed through the physiological source/sink terms (***Figs. 7b*** and ***8***) while advection plays a modest role (not shown, but its role as a nearshore and offshore source will be discussed below in the context of the annual budgets). For the total phytoplankton, the growth perturbation is largely compensated by changes in zooplankton grazing (***Fig. 7b***), a signature of propagation of the perturbation up the trophic chain. For all nearshore/offshore zooplankton classes, wind-induced perturbations in net growth are compensated by predation mortality (∼20–25%) and loss (75–80%), the latter being a mixture of excretion, egestion and natural mortality (about 45–50%, 35–40% and 10–20% of the loss, for both small and large zooplankton; 35–40%, 25–30% and 25–40% for predator zooplankton).

A careful examination of the small and large zooplankton budgets for the nearshore domain (***Fig. 8b–d***) indicates that small zooplankton fluxes are one order of magnitude smaller than the large zooplankton fluxes. We also note that small zooplankton predation mortality is only weakly affected by the winter wind perturbation. This confirms the nearshore dominance of the short food chain involving large species, both in terms of biomass (***Fig. 6***) and inter-size class fluxes (***Fig. 8***). In contrast, in the offshore region, the small and large zooplankton fluxes are of the same order and are equally affected by the winter wind perturbation. Offshore, the entire trophic web is modified by the perturbation. These points are explored in more detail in the next subsection.

#### Annual mean biological budgets

The effects of a winter wind perturbation are discussed using annual mean biological tracers budgets including advection and biological sources and sinks.

Nearshore (***Fig. 9***), a winter wind perturbation has a weak effect on the annual-mean budget, with a less than 10% flux increase (and a less than 7% biomass increase) in the early upwelling wind scenario compared to the late upwelling wind scenario. As described above, the nearshore ecosystem perturbation has a limited duration, roughly from January to March when winds differ (see the difference ratios over that period also given in ***Fig*** **. 9**), and the ecosystem (mainly the large plankton species) is stimulated by early upwelling winds. During the perturbation, the fluxes involving PL, ZL and ZP increase by more than 30% compared to the late upwelling case. Interestingly, the most sensitive food chain is PL to ZL to ZP, with a short-term increase of more than 40%. This chain is stimulated by a statistically significant increase of both new and regenerated production, in similar proportion. The remineralization loop is also enhanced, mainly via zooplankton losses to NH_4_ and PON/DON. This stimulation of the food chain has important consequences for the cross-shore export of biomass, which increases strikingly (∼50%) over the same period. On the other hand, the export of nutrients (nitrate, ammonium) is not affected.

Nearshore the modeled food chain involving small plankton species (PS and ZS) is weakly sensitive to the wind perturbation, as one would expect given the local dominance of large cells. The only notable perturbation of the nearshore fluxes concerns the grazing of PS by ZL in winter; this perturbation is unable to propagate up the small plankton chain because the ZL grazing pressure diverts it.

The effect of the wind perturbation on the offshore budget is noticeably larger than on the nearshore budget (***Fig. 9***). Most annual mean fluxes are >30% larger in the early upwelling wind case. Both small and large species are involved in the response to the wind perturbation: primary production, grazing, and predation fluxes increase by more than 30% in the early upwelling wind case (***Fig. 9***). The ecosystem stimulation by winter winds benefits ZP the most via predation. It is important to note that the stimulation of the entire food chain is primarily induced by a boost in regenerated production (30% increase for PS and 40% for PL), whereas the new production is only increased by 16% and 22% respectively. This offshore system perturbation stems largely from enhanced cross-shore advection of nearshore material: the physical transport is increased significantly (more than 30%) for biomass, PON and DON, and to a lesser extent for NO_3_ (26%).

Overall, this analysis demonstrates the importance of cross-shore transport of biomass and the recycling loop. Both processes strongly shape the ecosystem response to the winter wind perturbation. As it propagates offshore, the perturbation at low trophic levels is “recycled“ and increasingly migrates up the food chain.

**Figure 10:**
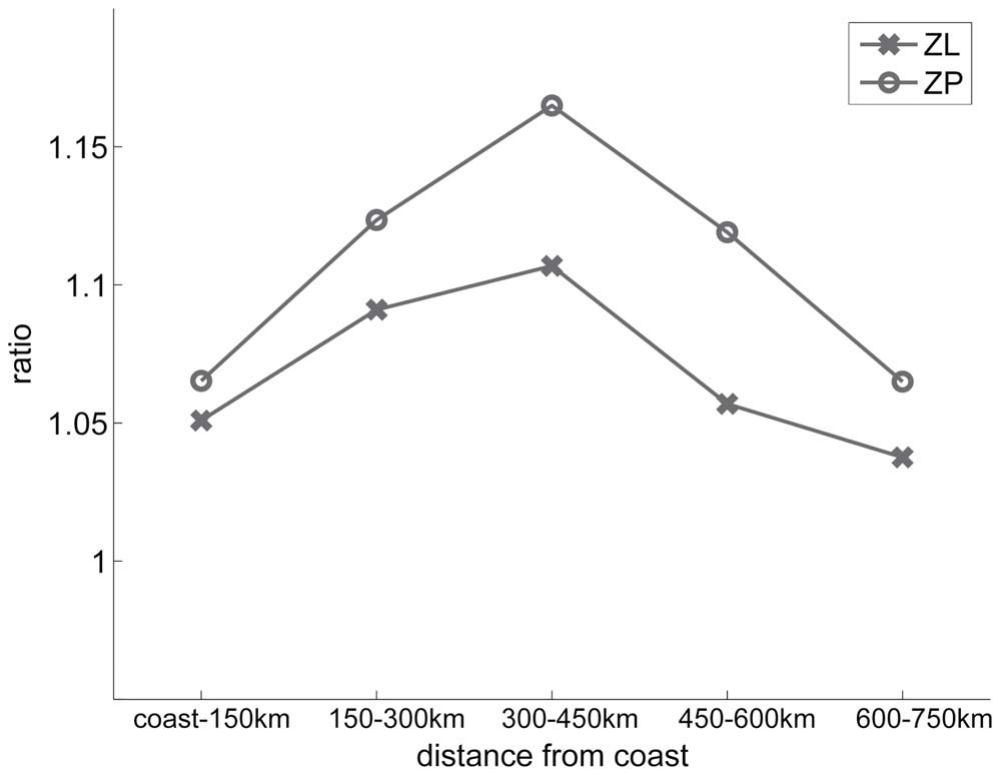
**Ratio of annual mean biomasses (see caption of Figure 9 for details)** **as a function of the distance from the coast, and computed in successive 150 km-wide boxes.** The ratios are given both for the large zooplankton and the zooplankton predator.

There is a limit to how far the perturbation moves offshore. Annual climatological mean ZL and ZP biomass ratios (between early- and late-upwelling scenarios) are the largest for the box situated 300 to 450 km offshore (***Fig. 10***). This maximum can be explained as follows: nearshore nutrients are abundant, and a reduction or increase of their concentration has less effect on production than farther offshore where nutrients are more strongly limiting (this was checked by computing the phytoplankton nutrient limitation terms). On the other hand, at a distance from the coast of the order of 1000 kilometers (see ***Fig. 10***), the perturbation is no longer felt because the enrichment of coastal origin plays a negligible role in the local ecosystem dynamics.

Several processes contribute to the cross-shore profile of the ecosystem response (***Fig. 10***): mean/eddy cross-shore advection, which propagates the anomaly offshore; alongshore advection, which advects the anomaly southward; and vertical export through sinking of particulate organic nitrogen (and possibly through subduction), which progressively attenuates the signal as it moves offshore. The representation of these processes and their relative importance depend to some degree on the model parameter settings. The horizontal grid resolution (15 km) is too coarse to simulate frontal processes realistically, so subduction is probably underestimated. The sinking velocities for particulate carbon are high (40 m/day see ***Table 2***) even though, despite large uncertainties [50], values around 10 m/day are often used in upwelling ecosystem studies [33], [34], [35], [51]. Our choice is conservative: it limits the offshore propagation of coastal perturbations, and avoids underestimation of both vertical sinking and subduction, which would have raised doubts about the cross-shore structure of the response to wind anomalies. We ensured that the cross-shore response of large and predator zooplankton was robust with respect to the parameters that control the recycling loop (nitrification, remineralization, and decomposition rates).

It is clear that eddies are important for the biogeochemical functioning of the California Current (see for instance [52]), and we have shown that offshore propagation of perturbations to the nearshore ecosystem is an important determinant of offshore ecosystem variability. We note, however, that the mesoscale activity in our numerical simulations does not change appreciably from one run to another (see Appendix S1 for a more detailed analysis).

## Conclusion

In this numerical study, we investigated the CCS ecosystem response to a time lag or lead in the onset of upwelling, as observed, for instance, in conjunction with NPGO variability. By analyzing the response in terms of structural changes (in biogeochemical tracer concentrations) and also in terms of dynamics (biogeochemical fluxes), we are able to clarify the mechanisms involved in driving the ecosystem response. The numerical approach relies on a biological and physical coupled model, forced by synthetic winds (derived from QuikSCAT climatology) that differ during winter (December to March). Despite some deficiencies, the model qualitatively represents the essential features of the CCS planktonic ecosystem (cross-shore gradient, seasonal cycle and depth distribution).

The intensity of early onset of upwelling winds exerts a major impact on the planktonic ecosystem, both in the nearshore region, but more importantly offshore (typically 300–500 km from the coast) where the perturbation is stronger, more persistent, and more complex. Nearshore, the ecosystem perturbation is undetectable in all biogeochemical fields after July. Before July, the effect of early upwelling winds simply arises from enhanced vertical nitrate fluxes that stimulate phytoplankton primary production and, in turn, zooplankton population growth. Overall, the changes in the nearshore ecosystem with respect to wind and upwelling changes (11% difference in Ekman transport) are modest, with less than a 7% difference in annual-average biomass between the two analyzed solutions (***Fig. 9***).

Offshore, where the biomass and biogeochemical tracer concentrations are much lower, the winter wind perturbation is felt most of the year and its relative amplitude is a factor 2 or more, greater than the nearshore. Most importantly, the highest trophic level (predator zooplankton) is by far the most impacted. The processes responsible for the offshore response are subtle and involve a combination of i) cross-shore transport which advects the coastal perturbation offshore at speeds consistent with an Ekman drift, ii) remineralization cycles which provide an increasingly large fraction of the nutrients available for primary production, and iii) relatively efficient transmission up the food chain through successive grazing/predation stages in which both small and large zooplankton participate.

**Figure 11:**
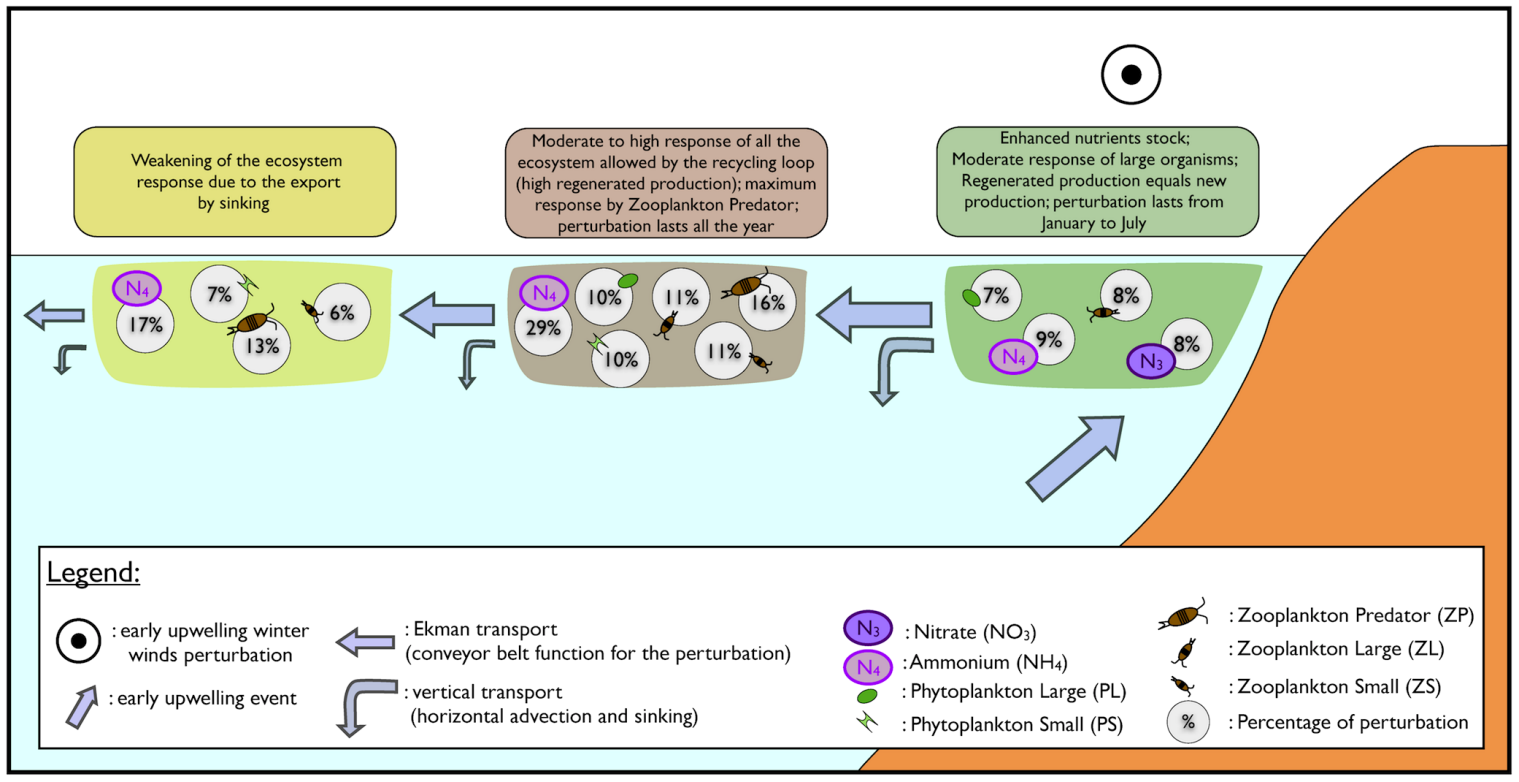
**Schematic view of the upwelling ecosystem response to a perturbation that consists in early coastal upwelling winter-winds.** This sketch illustrates the simultaneous propagation of the coastal upwelling/nitrogen perturbation cross-shore in space and bottom-up across the trophic chain. Relative changes in physical and biological processes affected by the perturbation are presented. 1) In the nearshore region (green box) the response is moderate and characterized by an increase of upwelled nitrates input and a modest increase of large organisms' biomass (mainly large phytoplankton PL and large zooplankton ZL). Moreover, in this region, the perturbation is no more detectable after July. 2) In the Offshore region (grey box), the response to the perturbation is larger and visible at all trophic levels and most importantly at the highest level (predator zooplankton ZP). This is induced by a wind-driven cross-shore transport of the coastal perturbation that recycles en route. We note a high perturbation of ammonium concentrations and the activation of the recycling loop. In this region, the perturbation lasts much longer and persists all the year. 3) In the far offshore region (yellow box), the perturbation is still non negligible with a main signature at the highest trophic level, despite a significant weakening of the ecosystem response because of vertical export by sinking.

There is thus a simultaneous propagation of the coastal upwelling/nitrogen perturbation in space (mainly cross-shore, with a modest contribution of eddies in our model) and from the bottom to the top of the trophic chain. During this propagation, which occurs over several months, the remineralization loop is very active on time scales of a few days. A total nitrogen (organic plus inorganic) perturbation climbs up the food chain while being transported offshore by the Ekman conveyor belt. A schematic view summarizes this propagation in ***Figure 11***. Leaks do exist (via sinking and alongshore advection by the California Current), but the typical length scale over which nearshore upwelled nitrogen exits the system is large enough for the perturbation to be felt hundreds of kilometers from the coast.

Our conclusions must be considered with caution as our study lacks realism in several respects. However, we believe they provide valuable original insight concerning ecosystem dynamics and response to large-scale wind anomalies. A horizontal model resolution of about 5 km would allow better realization of mesoscale eddies. We anticipate that at such resolution their effect would be increased: Gruber *et al.* [52] found that eddies reduce new primary production by as much as 70% in a region located 100 to 500 km off central California. Hence, better-resolved eddies would presumably further enhance the offshore propagation of the perturbation. In contrast, a more accurate representation of submesoscale frontal processes would lead to an increased nutrient export away from the euphotic zone that would probably attenuate the ecosystem perturbation more rapidly [52], [53]. A 1 km horizontal resolution simulation would be required to include both of these competing effects and determine their relative importance in the context of winter-wind perturbation propagation. Forcing variability at synoptic scales would also be important in that case; unfortunately, such forcing is not yet available. Lastly the ecosystem model used in this study included only minimal ecosystem structure. Hence, it provides a simplified view of the highly complex local ecosystem: it ignores size-structured relationships among organisms, size distribution of particles and aggregation dynamics known to play an important role in export production. It also includes a very simplified parameterization of higher trophic level predation and ignores iron limitation.

Higher trophic levels than those explicitly represented here tend to have marked preferences for the cross-shore location of their habitat. These preferences are seen as a tradeoff between food availability, optimal temperature for growth, predation level, and possibly other factors. More precisely, in the present system, food availability is higher nearshore, optimal temperatures for growth – while species-dependent – tend to be found offshore, and predation mortality (in particular at the larval stage) is higher in the macrozooplankton-rich nearshore waters. Several key CCS species such as the Pacific Sardine are found some distance away from the more eutrophic coastal zone. Our study suggests enhanced sensitivity of the offshore zone to winter wind anomalies as a possible new element to the habitat selection trade-off, with a late onset of upwelling having long-lasting detrimental effects. This would need to be tested with observations in which, unfortunately, other processes complicate the ecosystem response to a lag or lead in the upwelling onset timing. The late upwelling observed in 2005 (corresponding to a strong negative NPGO index) had profound consequences for the ecosystem [7]. In particular, off Oregon, *Mackas et al.* [54] reported nearshore zooplankton biomass anomalies persisting many months after the winds returned to normal. They hypothesized that the seasonal life history strategies of zooplankton may have affected their response to wind anomalies. A Lagrangian individual-based submodel would be needed to take zooplankton behavioral traits into account. It would also permit more elaborate analyses of nearshore versus offshore ecosystem sensitivity, through biogeochemical budgets along water parcel or animal trajectories.

## Supporting Information

Appendix S1 **In this appendix we give a detailed description of the cross-shore transport.** We quantify the eddy and mean flow contributions to the advective flux divergence in the tracer budgets: for nitrate concentration and for biomass.
(DOC)

## References

[1] Roemmich D, McGowan J (1995) Climatic warming and the decline of zooplankton in the California current. Science 267: 1324–1326. 17812604 10.1126/science.267.5202.1324

[2] Hare SR, Mantua NJ, Francis RC (1999) Inverse production regimes: Alaska and West Coast Pacific salmon. Fisheries 24 1: 6–14.

[3] Chavez FP, Ryan J, Lluch-Cota SE, Ñiquen M (2003) From anchovies to sardines and back: multidecadal change in the Pacific Ocean. Science 299: 217–221. 12522241 10.1126/science.1075880

[4] Legaard KR, Thomas AC (2006) Spatial patterns in seasonal and interannual variability of chlorophyll and sea surface temperature in the California Current. J Geophys Res 111: C06032.

[5] Di Lorenzo E, Schneider N, Cobb KM, Franks PJS, Chhak K, et al (2008) North Pacific Gyre Oscillation links ocean climate and ecosystem change. Geophys Res Lett 35 8: 1–6.

[6] Di Lorenzo E, Fiechter J, Schneider N, Bracco A, Miller AJ, et al (2009) Nutrient and salinity decadal variations in the central and eastern North Pacific. Geophys Res Lett 36 14: 2003–2008.

[7] Sydeman WJ, Thompson SA (2010) The California Current Integrated Ecosystem Assessment (IEA), Module II: Trends and Variability in Climate- Ecosystem State. NOAA Tech. Rep., NMFS SWFSC Environmental Research Division, (January), 1–59.

[8] Chenillat F, Rivière P, Capet X, Di Lorenzo E, Blanke B (2012) North Pacific Gyre Oscillation modulates seasonal timing and ecosystem functioning in the California Current upwelling system,. Geophys Res Lett 39: L01606.

[9] MacIsaac JJ, Dugdale RC, Barber RT, Blasco D (1985) Primary production cycle in an upwelling center. Deep-Sea Res II 32 5A: 503–529.

[10] Menge BA, Daley BA, Wheeler PA, Strub PT (1997) Rocky intertidal oceanography : An association between community structure and nearshore phytoplankton concentration. Limonol Oceanogr 42 1: 57–66.

[11] Snyder MA, Sloan LC, Diffenbaugh NS, Bell JL (2003) Future climate change and upwelling in the California Current. Geophys Res Lett 30 15: 1–4.

[12] Barth JA, Menge BA, Lubchenco J, Chan F, Bane JM, et al (2007) Delayed upwelling alters nearshore coastal ocean ecosystems in the northern California current. Proc Natl Acad Sci USA 104 10: 3719–24. 17360419 10.1073/pnas.0700462104 PMC1805484

[13] Dorman JG, Powell TM, Sydeman WJ, Bograd SJ (2011) Advection and starvation cause krill (*Euphausiapacifica*) decreases in 2005 Northern California coastal populations : Implications from a model study. Geophys Res Lett 38 4: 1–5.

[14] Ji R, Edwards M, Mackas DL, Runge JA, Thomas AC (2010) Marine plankton phenology and life history in a changing climate : current research and future directions. J Plankton Res 32 10: 1355–1368. 20824042 10.1093/plankt/fbq062 PMC2933132

[15] Landry MR (1977) A review of important concepts in the trophic organization of pelagic ecosystems. Helgoländer Wissenschaftliche Meeresuntersuchungen 30 1–4: 8–17.

[16] Botsford LW, Lawrence CA, Dever EP, Hastings A, Largier J (2006) Effects of variable winds on biological productivity on continental shelves in coastal upwelling systems. Deep-Sea Res II 53: 3116–3140.

[17] Shchepetkin AF, McWilliams JC (2005) The regional oceanic modeling system (ROMS) : a split-explicit, free-surface, topography following- coordinate oceanic model. Ocean Modell 9 4: 347–404.

[18] Marchesiello P, McWilliams JC, Shchepetkin AF (2003) Equilibrium Structure and Dynamics of the California Current System. J Phys Oceanogr 33 4: 753–783.

[19] Di Lorenzo E (2003) Seasonal dynamics of the surface circulation in the Southern California Current System. Deep-Sea Res II 50: 2371–2388.

[20] Capet X, Marchesiello P, McWilliams JC (2004) Upwelling response to coastal wind profiles. Geophys Res Lett 31 13: 1–4.

[21] Di Lorenzo E, Miller AJ, Schneider N, McWilliams JC (2005) The Warming of the California Current System: Dynamics and Ecosystem Implications. J Phys Oceanogr 35 3: 336–362.

[22] Capet X, Colas F, McWilliams JC, Penven P, Marchesiello P (2008) Eddies in eastern boundary subtropical upwelling systems, in Ocean Modeling in an Eddying Regime. Geophys Monogr Ser, vol. 177, edited by M. W. Hecht and H. Hasumi, pp. 131–147, AGU, Washington, D. C.

[23] Penven P, Marchesiello P, Debreu L, Lefèvre J (2007) Software tools for pre- and post-processing of oceanic regional simulations. Environmental Modelling & Software 23 5: 660–662.

[24] Woodson CB, Washburn L, Barth JA, Hoover DJ, Kirincich AR, et al (2009) Northern Monterey Bay upwelling shadow front: Observations of a coastally and surface-trapped buoyant plume. J Geophys Res: Oceans 114 12: 2156–2202.

[25] Castelao RM, Barth JM (2007) The Role of Wind Stress Curl in Jet Separation at a Cape. J Phys Oceanogr 37 11: 2652–2671.

[26] Schwing FB, O'Farrell M, Steger JM, Baltz K (1996) Coastal Upwelling indices west coast of North America. NOAA Tech. Rep., NMFS SWFSC NMFS SWFSC 231: 144p. NOAA, Seatlle, Wash.

[27] Bakun A (1973) Coastal upwelling indices, west coast of North America, 1946–71. U.S. Dep. Commer., NOAA Tech. Rep., NMFS SSRF-671, 103 p, NOAA, Seatlle, Wash.

[28] Kishi M, Kashiwai M, Ware D, Megrey B, Eslinger D, et al (2007) NEMURO - a lower trophic level model for the North Pacific marine ecosystem. Ecol Modell 202 1–2: 12–25.

[29] Rose K, Megrey B, Werner F, Ware D (2007) Calibration of the NEMURO nutrient–phytoplankton–zooplankton food web model to a coastal ecosystem: Evaluation of an automated calibration approach. Ecol Modell 202 1–2: 38–51.

[30] Wainwright T, Feinberg L, Hooff R, Peterson WT (2007) A comparison of two lower trophic models for the California Current System. Ecol Modell 202 1–2: 120–131.

[31] Li QP, Franks PJS, Landry MR, Goericke R, Taylor AG (2010) Modeling phytoplankton growth rates and chlorophyll to carbon ratios in California coastal and pelagic ecosystems. J Geophys Res 115 G4: 1–12.

[32] Li QP, Franks PJS, Landry MR (2011) Microzooplankton grazing dynamics : parameterizing grazing models with diluation experiment data from the California Current Ecosystem. Mar Ecol Prog Ser 438: 59–69.

[33] Powell TM, Lewis CVW, Curchitser E, Haidvogel DB, Hermann AJ, et al (2006) Results from a three-dimensional, nested biologicalphysical model of the California Current System and comparisons with statistics from satellite imagery. J Geophys Res 111 C7: 1–14.20411040

[34] Chai F, Dugdale R, Peng T, Wilkerson F, Barber R (2002) One dimensional ecosystem model of the equatorial Pacific upwelling system. Part I : model development and silicon and nitrogen cycle. Deep-Sea Res II 49 13–14: 2713–2745.

[35] Gruber N, Frenzel H, Doney SC, Marchesiello P, McWilliams JC, et al (2006) Eddy-resolving simulation of plankton ecosystem dynamics in the California Current System. Deep-Sea Res I 53 9: 1483–1516.

[36] Morozov AY (2010) Emergence of Holling type III zooplankton functional response: bringing together field evidence and mathematical modeling. Journal of theoretical biology 265 1: 45–54. 20406647 10.1016/j.jtbi.2010.04.016

[37] Gentleman W, Leising A, Frost B, Strom S, Murray J (2003) Functional responses for zooplankton feeding on multiple resources : a review of assumptions and biological dynamics. Deep-Sea Res II 50 22–26: 2847–2875.

[38] Masumoto Y, Sasaki H, Kagimoto T, Komori N, Ishida A, et al (2004) A fifty-year eddy-resolving simulation of the world ocean : Preliminary outcomes of OFES (OGCM for the Earth Simulator). Journal of Earth Simulator 1 April: 35–56.

[39] Sasaki H, Sasai Y, Kawahara M, Furuichi F, Araki A, et al.. (2004) A series of eddy -resolving ocean simulations in the world ocean : OFES (OGCM for the Earth Simulator) project. OCEAN'04, pp. 1535–1541.

[40] Sasaki H, Nonaka M, Masumoto Y, Sasai Y, Uehara H, et al.. (2006) An eddy-resolving hindcast simulation of the quasi-global ocean from 1950 to 2003 on the Earth Simulator. High Resolution Numerical Modelling of the Atmosphere and Ocean, edited by W. Ohfuchi and K. Hamilton, springer ed., New York.

[41] Chavez F (1989) Size distribution of phytoplankton in the central and eastern tropical Pacific. Global Biogeochem, Cycles 3 1: 27–35.

[42] Landry MR, Brown SL, Neveux J, Dupouy C, Blanchot J, et al (2003) Phytoplankton growth and microzooplankton grazing in high-nutrient, low-chlorophyll waters of the equatorial Pacific: Community and taxon-specific rate assessments from pigment and flow cytometric analyses. J Geophys Res 108 C12: 8142.

[43] Garcia HE, Locarnini RA, Boyer TP, Antonov JI (2006) World Ocean Atlas 2005. Vol. 4, Nutrients (phosphate, nitrate, silicate).

[44] Capet X, McWilliams JC, Molemaker MJ, Shchepetkin AF (2008) Mesoscale to submesoscale transition in the California Current System. Part I: Flow structure, eddy flux, and observational tests. J Phys Oceanogr 38: 29–43.

[45] Capet X, McWilliams JC, Molemaker MJ, Shchepetkin AF (2008) Mesoscale to submesoscale transition in the California Current System. Part II: Frontal processes. J Phys Oceanogr 38: 44–64.

[46] Vignudelli S, Kostianoy AG, Cipollini P, Benveniste J (2011) Coastal Altimetry, Springer-Verlag, Berlin, 578 pp.

[47] Cloern JE, Grenz C, Vidergar-Lucas L (1995) An empirical model of the phytoplankton chlorophyll : carbon ratio-the conversion factor between productivity and growth rate. Limnol Oceanogr 40 7: 1313–1321.

[48] Kruskal WH, Wallis WA (1972) Use of ranks in one-criterion variance analysis. J Am Stat Assoc 47: 583–621.

[49] Philander SG, Yoon JH (1982) Eastern boundary currents and coastal upwelling. J Phys Oceanogr 12: 862–879.

[50] Moriceau B, Gallinari M, Soetaert K (2007) Importance of particle formation to reconstructed water column biogenic silica fluxes. Global Biogeochem Cycles 21: GB3012.

[51] Koné V, Machu E, Penven P, Andersen V, Garçon V, et al (2005) Modeling the primary and secondary productions of the southern Benguela upwelling system: A comparative study through two biogeochemical models. Global Biogeochem Cycles 19: GB4021.

[52] Gruber N, Lachkar Z, Frenzel H, Marchesiello P, Münnich M, et al (2011) Eddy-induced reduction of biological production in eastern boundary upwelling systems,. Nature Geosci 4, 11: 787–792.

[53] Lathuilière C, Echevin V, Lévy M, Madec G (2010) On the role of mesoscale circulation on an idealized coastal upwelling ecosystem. J Geophys Res 115: C09018.

[54] Mackas DL, Peterson WT, Ohman MD, Lavaniegos BE (2006) Zooplankton anomalies in the California Current system before and during the warm ocean conditions of 2005. Geophys Res Lett 33: L22S07.

