# Supplementary material for: California Coastal Upwelling Onset Variability: Cross-Shore and Bottom-Up Propagation in the Planktonic Ecosystem
Source: PLoS One. 2013 May 15;8(5):e62281. doi: 10.1371/journal.pone.0062281 (PMC3655145; doi:10.1371/journal.pone.0062281)
Supplement: Appendix S1 — In this appendix we give a detailed description of the cross-shore transport. We quantify the eddy and mean flow contributions to the advective flux divergence in the tracer budgets: for nitrate concentration and for biomass. (DOC) [file pone.0062281.s001.doc]

**Appendix S1: characterization of the cross-shore transport**

The cross-shore advective fluxes bring 30% more organic and inorganic nitrogen to the offshore box in the early upwelling onset scenario compared to the late upwelling onset scenario. These differences are statistically significant when considered over the entire year, but the internal variability is too large to be more specific concerning their seasonality. We relate this to the fact that eddies play a considerable role in the offshore export of nutrients. The analysis of more subtle seasonal differences would require statistical averaging over a longer period (e.g., see [1]).

However, we can quantify the eddy contribution to the advective flux divergence in the tracer budgets. A mean-eddy decomposition of the cross-shore advection terms is performed following [2]. For a tracer whose concentration is noted *c* and a cross-shore velocity field *u*, this amounts to the pointwise computation of <*u’ . c’*>, averaged over the nearshore and offshore boxes. Primed quantities are deviations from the 12-year-averaged monthly mean climatology represented by <*u*> and <*c*>.

For nitrate concentration, the eddy fluxes represent a statistically significant fraction of the total advection and contribute to nearshore dilution and thus an offshore enrichment. In particular, in a late (early) upwelling wind scenario, the eddy fluxes represent 60% (67%) of the total advective flux nearshore and 52% (65%) of the total advective flux offshore. This weak sensitivity might stem from a slight increase in winter-spring EKE in the early upwelling wind scenario compared to a late upwelling wind scenario. However, the statistical significance of this EKE difference is unclear and we would need longer simulations with finer horizontal resolution to obtain robust statistics [3].

For the biomass, the eddy fluxes represent a smaller fraction of the total advective trends than the mean fluxes, and their importance varies with distance from the coast. Eddies play a minor role in the nearshore budget of phytoplankton and zooplankton: they account for about 10% of the total advection for both biological components. The role of eddies in the offshore budget of phytoplankton and zooplankton biomass is more important (about 20% of the total advective flux). The sign of their contribution is consistent with the diffusive nature of a mesoscale-driven transport: the eddies tend to increase the tracer concentrations offshore, at the expense of the nearshore which is richer in nitrogen. However, no statistically significant difference resulting from the wind perturbation was found in these percentages.

**References**

1. Colas F, Mc Williams JC, Capet X, Kurian J (2012) Heat balance and eddies in the Peru-Chile current system, Climate Dyn, 39 (1-2), 509–529.
2. Capet X, Colas F, McWilliams JC, Penven P, Marchesiello P (2008) Eddies in eastern boundary subtropical upwelling systems, in Ocean Modeling in an Eddying Regime. Geophys Monogr Ser, vol. 177, edited by M. W. Hecht and H. Hasumi, pp. 131–147, AGU, Washington, D. C.
3. Flierl G, McWilliams JC (1977) On the sampling requirements for measuring moments of eddy variability. J Mar Res, 35, 797–820.
